# Differential Structure of Inductive Proximity Sensor

**DOI:** 10.3390/s19092210

**Published:** 2019-05-13

**Authors:** Yi-Xin Guo, Cong Lai, Zhi-Biao Shao, Kai-Liang Xu, Ting Li

**Affiliations:** 1 School of Electronic and Information Engineering, Xi’an Jiaotong University, No. 28, Xianning West Road, Xi’an, Shaanxi 710049, China; laicong7@stu.xjtu.edu.cn (C.L.); agike@stu.xjtu.edu.cn (T.L.); 2 School of Automation and Information Engineering, Xi’an University of Technology, No. 5 South Jinhua Road, Xi’an, Shaanxi 710048, China; klxu@xaut.edu.cn

**Keywords:** inductive proximity sensor, displacement measurements, differential structure, linear approximation method, look-up table, airborne electronic equipment, small electric current pulse

## Abstract

The inductive proximity sensor (IPS) is applicable to displacement measurements in the aviation field due to its non-mechanical contact, safety, and durability. IPS can increase reliability of position detection and decrease maintenance cost of the system effectively in aircraft applications. Nevertheless, the specialty in the aviation field proposes many restrictions and requirements on the application of IPS, including the temperature drift effect of the resistance component of the IPS sensing coil. Moreover, reliability requirements of aircrafts restrict the use of computational-intensive algorithms and avoid the use of process control components. Furthermore, the environment of airborne electronic equipment restricts measurements driven by large current and proposes strict requirements on emission tests of radio frequency (RF) energy. For these reasons, a differential structured IPS measurement method is proposed in this paper. This measurement method inherits the numerical separation of the resistance and inductance components of the IPS sensing coil to improve the temperature adaptation of the IPS. The computational complexity is decreased by combining the dimension-reduced look-up table method to prevent the use of process control components. The proposed differential structured IPS is equipped with a differential structure of distant and nearby sensing coils to increase the detection accuracy. The small electric current pulse excitation decreases the RF energy emission. Verification results demonstrate that the differential structured IPS realizes the numerical decoupling calculation of the vector impedance of the sensing coil by using 61 look-up table units. The measuring sensitivity increased from 135.5 least significant bits (LSB)/0.10 mm of a single-sensing-coil structured IPS to 1201.4 LSB/0.10 mm, and the linear approximation distance error decreased from 99.376 μm to −3.240 μm. The proposed differential structured IPS method has evident comparative advantages compared with similar measuring techniques.

## 1. Introduction

Operation control processes from manufacturing [1,2], to chemical metallurgy [3], electric vehicles [4,5], biological medicine [6,7], and aerospace [8,9] require the detection of the position of mechanical moving components. Therefore, research on proximity sensors attracted wide attention in the industrial circle and became a major topic for sustainable development. The inductive proximity sensor (IPS) is widely used, especially in the aviation field with high requirements [8,10,11,12], due to its non-mechanical contact, safety, durability, reliability, and strong environmental suitability [8,13,14]. The demands for target location monitoring of transmission parts are increasing gradually with the development of aircraft toward larger size and more automation [15,16,17]. Monitoring points at the external side of aircraft are situated in severe environments, such as dusty environments, oily environments, spraying environments, freezing environments, acoustic optical disturbances, and low-temperature environments in cruising [18,19,20]. Thus, high requirements on the environmental adaptation of IPS are proposed in aviation [21,22,23]. The ambient temperature range for displacement detection in aviation is −55 °C–125 °C [24,25]. The relative position changes between sensing coil and target, which are detected by the IPS, are angular or straight variation [8,14]. The detection range of angular variation is generally 0–30°, which generally requires a measurement accuracy of 0.2–0.5° [26,27]. Meanwhile, the detection range of straight variation is generally 1–5 mm, which generally requires a measurement accuracy of 0.2–1.0 mm [28,29]. IPSs, which are improving continuously, are applied in various dynamic controls, such as landing gear [30,31], cabin doors [32], panels [33], and thrust reversers [34]. This type of sensor applies a full-closed structure [35,36,37] which can lower pollution effects [38]. This sensor plays an indispensable role in the detection of motion and positioning of airborne electro-mechanical components [29,37,39].

However, the general industrial-type IPS has difficulty in meeting index requirements of the aviation field due to specificity of the space environment.

The requirement of aviation reliability restricts the use of computational-intensive algorithms and avoids the use of process control components, such as micro-controller units (MCUs) and digital signal processors (DSPs) [29,40,41]. Since these types of components contain abundant random-access memory units [42,43], error of any logic state may cause control failure [44,45]. The monitoring points of motor displacement on aircraft are increasing continuously and exhibit causality relationships [46,47]. The process control component increases the probability of the entire system failing [48]. The common high-accuracy industrial IPS, which measures inductance on the basis of a phase detection method, uses process control components similar to MCUs or DSPs [49]. Thus, this type of sensor has difficulty in assuring the global reliability performance of aircraft (mean time between failure (MTBF) > 200,000 h) [29,50,51].

Environmental condition requirements of airborne electronic equipment propose stricter test standards of radio frequency (RF) energy emission and a wider frequency range than similar general industrial standards of electromagnetic compatibility [24,25]. General industrial IPSs cannot meet the test standards [52]. For example, the magnetic resistance proximity sensor uses a large-current (>500 mA) driving sensing coil and produces the digital output related to distance on the basis of changes in magnetic resistance, and it cannot measure the distance accurately [53,54]. The open magnetic circuit in the sensing coil has electromagnetic oscillation and, thus, cannot meet restrictions on RF energy emission in aviation [29,41,55].

The IPS sensing coil is formed by copper wire winding (oxygen-free copper) and has a resistance temperature coefficient of 3950 ppm/°C. The operating temperature range of airborne electronic equipment is −55 °C–125 °C [24,25], and the total temperature drift of the corresponding resistance component is higher than 71%, thereby resulting in a large distance-measuring error [29,56].

General IPS digital measurement methods apply sine excitation to the sensing coil and then sample the responses. It separates the resistance and inductance components of the IPS sensing coil on the basis of differences in phase and amplitude of the responses, and determines the measured distance by contrasting the setting of the threshold or look-up table [57,58]. The measurement accuracy can be improved by inhibiting the resistance temperature drift of the sensing coil, which can be output quantitatively [59]. General IPS digital measurement methods need MCU or DSP components [56,57], which consume large amounts of power and hardware, due to the complicated control and calculation processes. The MTBF performance has difficulty in meeting the standard in aviation [29,41,56].

General IPS analog measurement methods apply step input to the sensing coil and then compare the response value at a fixed moment and a given threshold to obtain the distance [60,61]. This method has a simple circuit, and can reduce the resistance temperature drift through a customization of thermal resistor compensation [60,62]. However, this method has a narrow working temperature range, a low measurement accuracy [63], and temperature–time drifts of other analog components, which restrict its stability [29,64,65]. There are also switchable highly sensitive inductance sensing methods (which are used in IPS sensors) with temperature compensation without a temperature sensor using quartz crystals which have low drift and high stability [66,67].

A traditional IPS analog measurement method applied in the aviation field [41] provides period excitation to the sensing coil with a voltage-controlled oscillator (OSC) and converts the response current into the output voltage proportional to inductance value through the analog circuit. Thereafter, the output voltage is compared with the set threshold to obtain the pulse output signal representing distance. The narrow pulse of the OSC locks the displacement signal output by launching the flip-flop to obtain stable level output after filtering. The distance threshold can be set by selecting parameters of analog components. This IPS in aviation can output qualitatively, but the instabilities brought by the long-term drifts of analog components have to be compensated and adjusted periodically.

Leons and Yaghoubian proposed a digital IPS measurement method for aviation [42,68]. This method uses a field-programmable gate array to apply a step input on the sensing coil by controlling a digital-to-analog converter, implementing high-speed sampling of response waveforms in a certain period, and accomplishing the calculation of the integral value of response waveforms. A one-dimensional look-up table is set up on the basis of the relationship between the integral value of response waveforms and the measured distances for obtaining the distance. The look-up table simplifies the calculation and saves the use of process control units. The results demonstrated that 0–5-mm distance measurements under room temperature can reach a measurement resolution of ±0.1 mm. This digital IPS measurement method fails to separate the resistance and inductance components of the IPS sensing coil. This previous study also concluded that “the characterization hardware is generic enough to be reused for characterization with changes in temperature”. However, relevant studies [29,56] based on detailed analysis of test data reported that measurement errors caused by environmental temperature are considerably greater than the 0.1-mm resolution.

Previous studies proposed an analog–digital mixed IPS measurement method [29]. This method excites the sensing coil via a narrow-pulse signal and gains the sampling vector to research the two-dimensional look-up table through sampling of response waveforms twice at fixed moments for obtaining the measured distance. The resistance and inductance components of the IPS sensing coil are separated and calculated through the decoupling algorithm, and effects of resistance temperature drift are inhibited. The look-up table method decreases the complexity of computation and avoids the use of process control units. The size of the look-up table is decreased significantly by compressing the two-dimensional look-up table. On this basis, a dimension-reduced look-up table method was proposed [56]. This method decreases the complexity of online computation, determines structural advantages of the look-up table method, reduces the size of the look-up table significantly, and increases the accuracy and range of the distance measurement. The results of verification and usage [56] showed that the dimension-reduced look-up table method has superior performance to similar IPSs.

The dimension-reduced look-up table method decreases the complexity of online computation, reduces the look-up table scale significantly, and increases the reliability and potential of performance promotion. Further analysis demonstrated that the variation range of sample values at a fixed moment with distance is limited due to the nonlinear distribution relationship between the inductance component of the sensing coil and distance. This variation range is approximately 27% of the dynamic range of analog–digital converters (ADCs). The low actual conversion utilization restricts the expected increase in measurement resolution and improvement in detection accuracy.

For this reason, a differential structured IPS is proposed herein on the basis of previous studies. The response circuit of this differential structured IPS includes the symmetric distant and nearby sensing coils. The inductance component responses of the two sensing coils are different. The responses of two symmetric circuits are differenced and amplified by an instrument amplifier (INA) before sampling to increase the sensitivity of response. This method inherits the linear approximation and the look-up table methods in the dimension-reduced look-up table method and maintains the low complexity of computation. The differential structured IPS increases the proportion of sample values at two fixed moments in the dynamic range of the ADC and the effective resolution of distance measurements. Moreover, this type of sensor can strengthen the interference resistance of the system and the length adaptation of the connection cable. The designed differential structured and dimension-reduced IPS realizes numerical decoupling calculations of vector impedance of the sensing coil by using 61 look-up table units. The performance verification of the differential structured IPS demonstrates that the measurement resolution increases from 135.5 least significant bits (LSB)/0.10 mm to 1201.4 LSB/0.10 mm compared with that of single-sensing-coil structured IPS, and the interpolation error decreases from 99.376 μm to −3.240 μm. Compared to similar IPS methods, the improved differential structured and dimension-reduced IPS possesses evident advantages in measurement accuracy.

## 2. Structure of Sensor Characteristics

**Figure 1:**
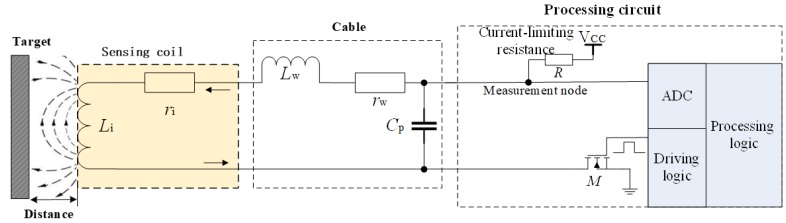
Block diagram of single-sensor-coil structured inductive proximity sensor (IPS).

**Figure 2:**
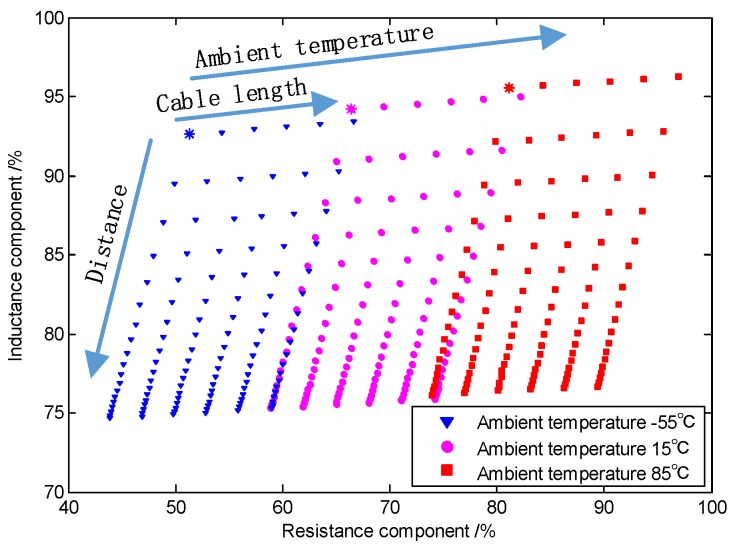
Relationships between vector impedance of the response circuit and environmental factors.

The sensing coil is formed by the iron core that is wound with oxygen-free copper wires, and the local open magnetic field changes with the variation in distance between the sensing coil and the target. These characteristics are manifested by the sensitivity of the inductance component of the IPS sensing coil. The processing circuit applies excitation to the sensing coil, detects the response at the measurement node, calculates the inductance parameters of the sensing coil, and outputs the distance between the sensing coil and the target. The sensing coil is equivalent to a series circuit of inductance component *L*_i_ and resistance component *r*_i_, as shown in Figure 1. The inductance component *L*_i_ changes slightly with the variation in temperature and is very sensitive to distance. Changes in the resistance component with distance can be neglected, but the temperature drift cannot be ignored. The relationships of the vector impedance of the sensing coil with distance, temperature, and cable length are shown in Figure 2.

The IPS for aircraft generally requires 0.2–1.0 mm of displacement measurement accuracy in the detection range of 1–5 mm. The sensing coil is wound with the oxygen-free copper wire, and its resistance temperature coefficient is 3950 ppm/°C. In the operating ambient temperature range of −55 °C–125 °C, the total resistance temperature drift is 71%, which reduces the measurement accuracy of the IPS. For this reason, reducing the disturbance of the temperature drift is the key point to improve the precision of IPS.

**Figure 3:**
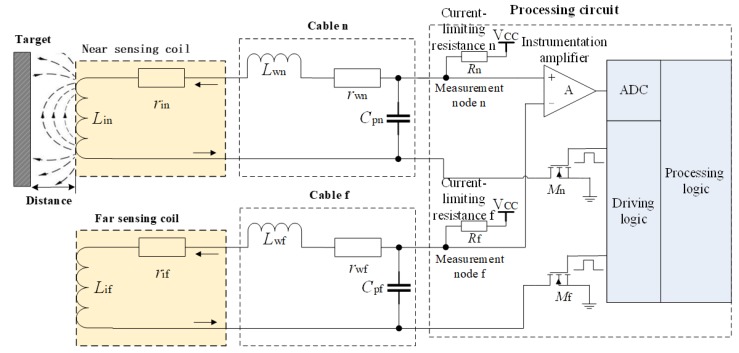
Block diagram of differential structured IPS.

The structure of the differential structured IPS based on previous studies on a single-sensing-coil structured IPS is shown in Figure 3. The differential structured IPS is composed of two sensing coils that form the two symmetric response circuits. The responses of the two symmetric response circuits are differenced and amplified by the INA before the sampling of the ADC. Given that the nearby sensing coil is close to the target, its inductance component *L*_in_ is highly sensitive to the distance. The distant sensing coil is between the nearby sensing coil and the processing circuit, is relatively far from the target, and has an inductance component *L*_if_ that is less sensitive to the distance. The resistance components of nearby and distant sensing coils (*r*_in_ and *r*_if_) impose the same effects on environmental temperature. The typical parameters of IPS are as follows: the inductance component *L*_in_ of nearby sensing coil changes between 4.9 and 9.5 mH, the sensitivity of *L*_if_ is decreased significantly and is set to 10% of *L*_in_, and the resistance components (*r*_in_ and *r*_if_) under 20 °C are approximately 13.2 Ω with a temperature drift of 3950 ppm/°C. Furthermore, *r*_in_ and *r*_if_ change in the range of 9.3–18.7 Ω in the environmental temperature range of −55 °C–125 °C. In consideration of the manufacturing deviation of sensing coils, the vector impedances of the distant sensing coil are set as 95% of that of the nearby sensing coil. In consideration of the current limiting conditions (smaller than 20 mA), the current-limiting resistances of the response circuits (*R*_n_ and *R*_f_) are chosen as 300 Ω.

The nearby and distant sensing coils form symmetric response circuits. The driving logic produces periodic and synchronous excitations to the sensing coils through the switching transistors *M*_n_ and *M*_f_. The responses on two symmetric measurement nodes are differenced and amplified through the INA. The processing circuit samples the differential response voltage through the ADC, gains the characteristics of response waveforms, and calculates the measured distance. The nearby and distant sensing coils are connected with the processing circuit through equivalent-length cables. The integrated IPS cable is very short, and the distribution parameters of the cables can be neglected. The non-integrated IPS is composed of multiple sensing units, which are connected to a multi-channel processing circuit through cables. The connection cables are equivalent to a connection in series of the equivalent resistance *r*_w_ and equivalent distributed inductance *L*_w_, which is connected in parallel with the equivalent distributed capacitance *C*_p_. The measurement results show that *L*_w_ = 457 nH/m, *r*_w_ = 84.7 pF/m, and *C*_p_ = 72.4 pF/m for typical unshielded twisted pair cables. The equivalent distributed parameters of cables are considerably smaller than the vector impedance of the sensing coil, and the influences of temperature drift can be neglected. The special cable is made of a silver-coated copper wire covered by a Teflon insulating layer, and presents excellent temperature stability. Similar to the resistance component of the sensing coil, the equivalent resistance of cable can be calculated and separated by the circuit equation, which inhibits the influence of the temperature drift of cable.

## 3. Analysis of Sensor Characteristics

**Figure 4:**
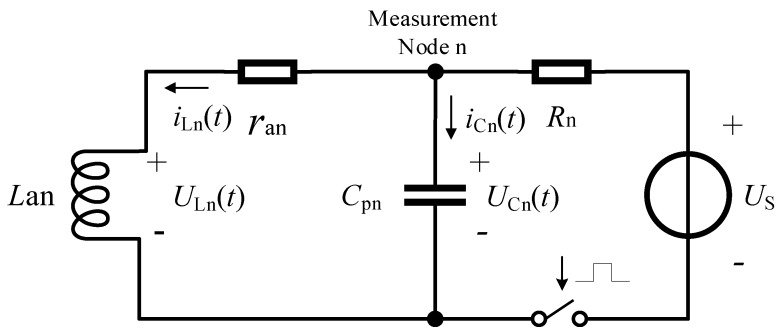
Equivalent circuit of the nearby sensing coil.

The equivalent circuits corresponding to the nearby and distant sensing coils are similar. The equivalent circuit of the nearby sensing coil is shown in Figure 4. The parameters can be combined into *L*_in_ + *L*_wn_ = *L*_an_ and *r*_in_ + *r*_wn_ = *r*_an_, as the inductance component *L*_in_, the resistance component *r*_in_, the equivalent distributed inductance of the cable *L*_wn_, and the equivalent resistance *r*_wn_ compose a series circuit. *U*_s_ is the power source of driving voltage, and the driving logic switches the switching transistor periodically to realize step input to the circuit. In consideration of *L*_an_ and the equivalent distributed capacitance of cable *C*_pn_, the equivalent circuit is a second-order inertia system. *U*_Cn_(*t*) represents the voltage response waveform on the measurement node, which is also the voltage response of *C*_pn_. *U*_Ln_(*t*) represents the response voltage of *L*_an_, while *i*_Ln_(*t*) and *i*_Cn_(*t*) are currents flowing through *L*_an_ and *C*_pn_.

The equivalent circuit is constrained by the second-order differential equation. The circuit loop from *U*_s_^+^ to *U*_s_^−^ through *R*_n_, *r*_an_, and *L*_an_ and the current loop from *C*_pn_^+^ (*U*_cn_^+^) to *C*_pn_^−^ (*U*_cn_^−^) through *r*_an_ and *L*_an_ correspond to two independent Kirchhoff’s voltage law equations given by

(1) $$\left\{ \begin{array}{l} R_{n}(i_{\mathrm{Cn}}+i_{\mathrm{Ln}})+U_{\mathrm{Cn}}=U_{S}\\ -U_{\mathrm{Cn}}+r_{\mathrm{an}}i_{\mathrm{Ln}}+U_{\mathrm{Ln}}=0 \end{array}\right..$$

By substituting the voltage–current relationships (VCRs) of the energy storage units *C*_pn_ and *L*_an_ into Equation (1), the differential equations of the equivalent circuit are

(2) $$\left\{ \begin{array}{l} R_{n}(C_{p}{}_{n}\frac{dU_{\mathrm{Cn}}}{dt}+i_{\mathrm{Ln}})+U_{\mathrm{Cn}}=U_{S}\\ -U_{\mathrm{Cn}}+r_{\mathrm{an}}i_{\mathrm{Ln}}+L_{\mathrm{an}}\frac{di_{Ln}}{dt}=0 \end{array}\right.,$$

where the response voltage *U*_Cn_(*t*) and inductance current *i*_Ln_(*t*) are variables. Then, *i*_Ln_(*t*) is eliminated, and the differential equation with *U*_Cn_(*t*) only is

(3) $$R_{n}C_{\mathrm{pn}}L_{\mathrm{an}}\frac{d^{2}U_{\mathrm{Cn}}}{dt^{2}}+(R_{n}C_{\mathrm{pn}}r_{\mathrm{an}}+L_{\mathrm{an}})\frac{dU_{\mathrm{Cn}}}{dt}+(R_{n}+r_{\mathrm{an}})U_{\mathrm{Cn}}=r_{\mathrm{an}}U_{S}.$$

The characteristic root of this equation is

(4) $$p_{n1,2}=\frac{-R_{n}C_{\mathrm{pn}}r_{\mathrm{an}}-L_{\mathrm{an}}\pm \sqrt{{\left(R_{n}C_{\mathrm{pn}}r_{\mathrm{an}}+L_{\mathrm{an}}\right)}^{2}-4R_{n}C_{\mathrm{pn}}L_{\mathrm{an}}(R_{n}+r_{\mathrm{an}})}}{2R_{n}C_{\mathrm{pn}}L_{\mathrm{an}}}.$$

The form of characteristic roots determines the characteristics of the response waveform. When the characteristic roots are two conjugate complex numbers, the second-order inertial system is in the underdamped case, and the response waveform attenuates in the oscillation mode. When the characteristic roots are two real numbers, the second-order inertial system is in the overdamped case. The response waveform at the measurement node is increased (caused by the capacity charge), and then decreased (caused by the inductance charge), and finally converged to the direct voltage controlled by the ratio of *R*_n_ to *r*_an_. The response waveform has one and only one inflection point. When the characteristic roots are two equal real numbers, the second-order inertial system is in the critical state.

The processing method of sampling several points on the response waveform and searching the look-up table for the parameters of electronic components in the testing circuit requires that the equation set of the look-up table has one and only one solution to the sampling vector input. Moreover, this method requires that the response waveform of the equation set is (locally) monotonous. The response waveform has one and only one inflection point when the second-order inertial system is in the overdamped case. The response characteristics before the inflection point are formed by the equivalent distributed capacitance charging in the equivalent circuit. This capacitance is a cable distribution parameter, and its alternating current (AC) impedance is considerably smaller than that of the sensing coil. Thus, as the charging approaches to saturation, the influences of the AC impedance on the response waveform before the inflection point decrease quickly. Main characteristics of the response waveform after the inflection point are constrained by the inductance charging of the sensing coil.

Parameter ranges of the sensing coil and cable are controlled to ensure the overdamped case of the corresponding inertial system. Sampling and look-up table calculations are carried out by the response waveform after the inflection point. Relevant parameters have to meet

(5) $${\left(R_{n}C_{\mathrm{pn}}r_{\mathrm{an}}+L_{\mathrm{an}}\right)}^{2}-4R_{n}C_{\mathrm{pn}}L_{\mathrm{an}}(R_{n}+r_{\mathrm{an}})>0.$$

**Figure 5:**
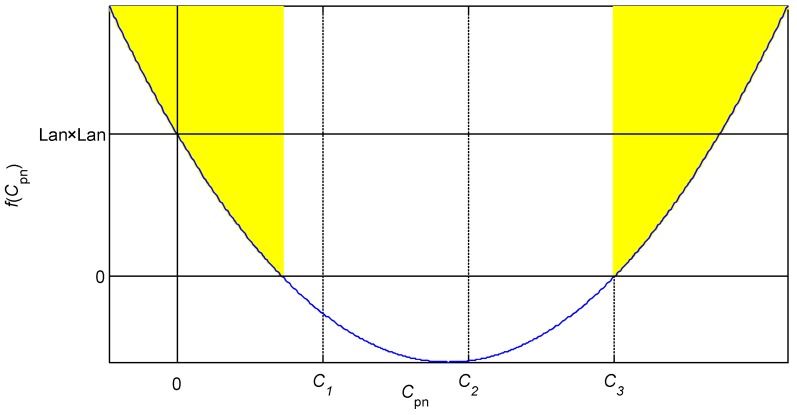
Damped cases of the system determined by the value of *f*(*C*_pn_).

The equivalent distributed capacitance of cable is the main factor that causes second-order inertial behavior of the response waveform. The response waveform is close to the one-order system and is far from the underdamped case when the cable is short. Therefore, the length of cable is the major factor that determines whether the constraint condition is true. The abovementioned constraint relationship is the relationship between the cable equivalent distributed capacitance and the damped case of the corresponding inertial system.

(6) $$f(C_{\mathrm{pn}})={\left(R_{n}r_{\mathrm{an}}\right)}^{2}C_{\mathrm{pn}}{}^{2}-2R_{n}L_{\mathrm{an}}(r_{\mathrm{an}}+2R_{n})C_{\mathrm{pn}}+L_{\mathrm{an}}{}^{2}>0,$$

where all parameters are the physical parameters in positive real numbers. Therefore, the curve of the quadratic function *f*(*C*_pn_) is a parabola opening upward and passing through (0, *L*_an_^2^), and the symmetric axial coordinate is a positive number, as shown in Figure 5.

For the two shaded regions in Figure 5, the value of *f*(*C*_pn_) is positive when *C*_pn_ is smaller than the constant *C*_1_ or higher than the constant *C*_3_, and the second-order inertial system is in the overdamped case. For the blank region in Figure 5, the value of *f*(*C*_pn_) is negative when *C*_pn_ is higher than the constant *C*_1_ and smaller than the constant *C*_3_, and the inertial system is in the underdamped case. The value of *f*(*C*_pn_) is 0 when *C*_pn_ is equal to the constant *C*_1_ or *C*_3_, and the inertial system is in the critical damped case. The values of constants in Figure 5 can be calculated by the following equation:

(7) $$\left\{ \begin{array}{l} C_{1}=\frac{L_{\mathrm{an}}(r_{\mathrm{an}}+2R_{n})-2L_{\mathrm{an}}\sqrt{R_{n}{}^{2}+r_{\mathrm{an}}R_{n}}}{R_{n}r_{\mathrm{an}}{}^{2}}\\ C_{2}=\frac{L_{\mathrm{an}}(r_{\mathrm{an}}+2R_{n})}{R_{n}r_{\mathrm{an}}{}^{2}}\\ C_{3}=\frac{L_{\mathrm{an}}(r_{\mathrm{an}}+2R_{n})+2L_{\mathrm{an}}\sqrt{R_{n}{}^{2}+r_{\mathrm{an}}R_{n}}}{R_{n}r_{\mathrm{an}}{}^{2}} \end{array}\right..$$

The IPS environment is set to room temperature (*r*_in_ = 13.2 Ω), while the typical distance threshold is 3.5 mm (*L*_in_ = 5.1 mH), and the cable length is 100 m. The calculated parameters are as follows: equivalent inductance *L*_an_ = 5.15 mH, equivalent distributed capacitance *C*_pn_ = 7.24 nF, and equivalent resistance *r*_an_ = 21.67 Ω. In the current-limiting condition, resistance *R*_n_ is 300 Ω, and then the constants are *C*_1_ = 13.80 nF, *C*_2_ = 22.71 μF, and *C*_3_ = 45.40 μF. Considering that *C*_pn_ < *C*_1_, the second-order inertial system is in the overdamped case when the cable is 100 m. The cable length is generally shorter than 20 m. Thus, the second-order inertial system is reliably in the overdamped case.

From the damped case of the equation, the solution to the equation set can be determined by

(8) $$\left\{ \begin{array}{l} U_{\mathrm{Cn}}(t)=A_{n1}e^{p_{n1}t}+A_{n2}e^{p_{n2}t}+Q(t)\\ p_{n1,2}=\frac{-R_{n}C_{\mathrm{pn}}r_{\mathrm{an}}-L_{\mathrm{an}}\pm \sqrt{{\left(R_{n}C_{\mathrm{pn}}r_{\mathrm{an}}+L_{\mathrm{an}}\right)}^{2}-4R_{n}C_{\mathrm{pn}}L_{\mathrm{an}}(R_{n}+r_{\mathrm{an}})}}{2R_{n}C_{\mathrm{pn}}L_{\mathrm{an}}}\\ {\left(R_{n}C_{\mathrm{pn}}r_{\mathrm{an}}+L_{\mathrm{an}}\right)}^{2}-4R_{n}C_{\mathrm{pn}}L_{\mathrm{an}}(R_{n}+r_{\mathrm{an}})>0\\ t>0 \end{array}\right.,$$

where the constants of general solution *A*_n1_ and *A*_n2_ can be calculated by the initial conditions, and the special solution Q(t) can be calculated through the input signal.

The driving logic controls the state switching transistor and applies periodic step input onto the sensing coil. Before the rising edge of step input, all energy storage components are discharged completely after full delay. Therefore, the rising edge of step input produces a zero-state response. The equivalent circuit indicates that the total equivalent inductance *L*_an_ is considered a short circuit, and the cable equivalent distributed capacitance *C*_pn_ is considered an open circuit when the step input is set up (corresponding to the 0^+^ moment). Voltage at the measurement node *U*_Cn_ is only influenced by the division of the total equivalent resistance *r*_an_ and the current-limiting resistance *R*_n_ over the high voltage of the step input *U*_S_. The special solution of the equation is

(9) $$Q(t)=\frac{r_{\mathrm{an}}}{r_{\mathrm{an}}+R_{n}}U_{S}.$$

The zero-state initial condition of the equivalent circuit indicates that the total equivalent inductance *L*_an_ is discharged completely, and the current in inductance is 0. The cable equivalent distributed capacitance *C*_pn_ is discharged completely, and the voltage on the capacitance is 0. By combining VCRs, the zero-state constraints of the system are

(10) $$\left\{ \begin{array}{l} U_{C}{}_{n}(0^{+})=0\\ i_{L}{}_{n}(0^{+})=0 \end{array}\right..$$

By substituting the initial condition into *U*_Cn_(*t*) in Equation (8), the constraint of *A*_n1_ and *A*_n2_ is

(11) $$U_{\mathrm{Cn}}(0^{+})=A_{n1}+A_{n2}+\frac{r_{\mathrm{na}}}{r_{\mathrm{an}}+R_{n}}U_{S}=0.$$

The first equation in Equation (2) is organized and substituted into the initial condition as follows:

(12) $$\frac{dU_{\mathrm{Cn}}}{dt}|\begin{array}{l} t=0^{+} \end{array}=\frac{U_{S}-U_{\mathrm{Cn}}(0^{+})-R_{n}i_{\mathrm{Ln}}(0^{+})}{R_{n}C_{\mathrm{pn}}}=\frac{U_{S}}{R_{n}C_{\mathrm{pn}}}.$$

The derivative of Equation (8) is calculated and brought into the initial conditions as follows:

(13) $$\frac{dU_{\mathrm{Cn}}}{dt}|\begin{array}{l} t=0^{+} \end{array}=p_{n1}A_{n1}+p_{n2}A_{n2}.$$

By combining Equations (12) and (13), another constraint of *A*_n1_ and *A*_n2_ is

(14) $$\frac{U_{S}}{R_{n}C_{\mathrm{pn}}}=p_{n1}A_{n1}+p_{n2}A_{n2}.$$

Equations (11) and (14) form two independent constraints of *A*_n1_ and *A*_n2_, and the constants of the general solution can be solved. The solution of the inertial system is

(15) $$\left\{ \begin{array}{l} U_{\mathrm{Cn}}(t)=A_{n1}e^{p_{n1}t}+A_{n2}e^{p_{n2}t}+\frac{r_{\mathrm{an}}}{r_{\mathrm{an}}+R_{n}}U_{S}\\ A_{n1}=\frac{\frac{1}{R_{n}C_{\mathrm{pn}}}+p_{n2}\frac{r_{\mathrm{an}}}{R_{n}+r_{\mathrm{an}}}}{p_{n1}-p_{n2}}U_{S}\\ A_{n2}=\frac{\frac{1}{R_{n}C_{\mathrm{pn}}}+p_{n1}\frac{r_{\mathrm{an}}}{R_{n}+r_{\mathrm{an}}}}{p_{n2}-p_{n1}}U_{S}\\ p_{n1}=\frac{-R_{n}C_{\mathrm{pn}}r_{\mathrm{an}}-L_{\mathrm{an}}+\sqrt{{\left(R_{n}C_{\mathrm{pn}}r_{\mathrm{an}}+L_{\mathrm{an}}\right)}^{2}-4R_{n}C_{\mathrm{pn}}L_{\mathrm{an}}(R_{n}+r_{\mathrm{an}})}}{2R_{n}C_{\mathrm{pn}}L_{\mathrm{an}}}\\ p_{n2}=\frac{-R_{n}C_{\mathrm{pn}}r_{\mathrm{an}}-L_{\mathrm{an}}-\sqrt{{\left(R_{n}C_{\mathrm{pn}}r_{\mathrm{an}}+L_{\mathrm{an}}\right)}^{2}-4R_{n}C_{\mathrm{pn}}L_{\mathrm{an}}(R_{n}+r_{\mathrm{an}})}}{2R_{n}C_{\mathrm{pn}}L_{\mathrm{an}}}\\ {\left(R_{n}C_{\mathrm{pn}}r_{\mathrm{an}}+L_{\mathrm{an}}\right)}^{2}-4R_{n}C_{\mathrm{pn}}L_{\mathrm{an}}(R_{n}+r_{\mathrm{an}})>0\\ t>0 \end{array}\right.,$$

where *U*_S_ is the high level of the step input.

**Figure 6:**
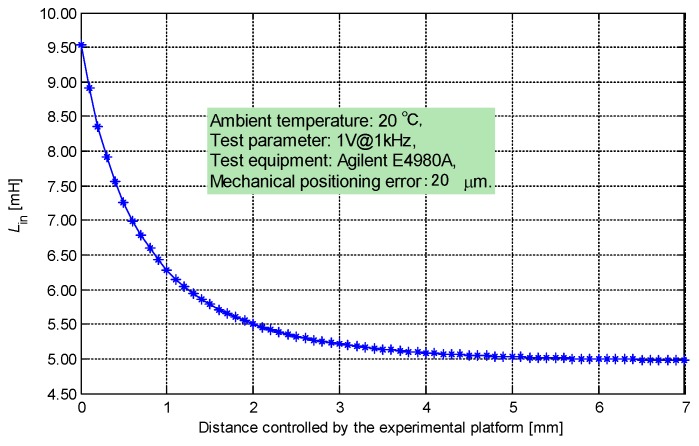
Calibration of distance vs. *L*.

**Figure 7:**
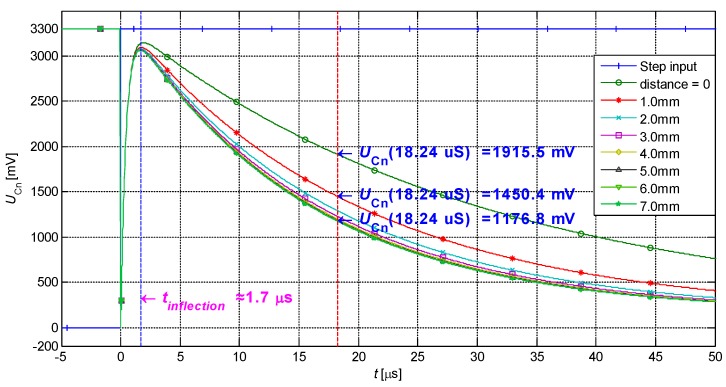
Response waveforms of the inertial system.

The relationship between the inductance component of the nearby sensing coil and the distance is obtained through calibration, as shown in Figure 6. The response waveforms (20 °C, cable length = 10 m) of multiple distances in the overdamped case when *U*_S_ = 3.3 V are drawn, as shown in Figure 7. Every response after the zero point has one and only one inflection point. The inflection point is constrained by the following equation:

(16) $$\left\{ \begin{array}{l} \frac{dU_{\mathrm{Cn}}(t)}{dt}=p_{n1}A_{n1}e^{p_{n1}t}+p_{n2}A_{n2}e^{p_{n2}t}=0\Rightarrow t=\frac{\ln(-p_{n2}A_{n2}/p_{n1}A_{n1})}{p_{n1}-p_{n2}}\\ {\left(R_{n}C_{\mathrm{pn}}r_{\mathrm{an}}+L_{\mathrm{an}}\right)}^{2}-4R_{n}C_{\mathrm{pn}}L_{\mathrm{an}}(R_{n}+r_{\mathrm{an}})>0\\ t>0 \end{array}\right..$$

These response waveforms present the monotone decreasing distribution after its inflection point. The response characteristics in the time domain reflect the distance between the sensing coil and target and form a functional relationship.

## 4. Algorithm Implementation of the Differential Structure

The voltage of the response loop of the nearby sensing coil at the measurement node is collected through the ADC, through which parameters of the sensing coil can be calculated, and the distance can be obtained. The single-sensing-coil structured IPS, which is designed on the basis of early studies, is produced in large quantities. Nevertheless, this single-sensing-coil structured IPS has a prominent disadvantage. Specifically, the voltage of the response waveform at the sampling moment changes with the distance, and the variation range accounts for a small proportion in the dynamic range of the ADC, thereby restricting sensitivity of the sensor. This disadvantage is mainly manifested in the following aspects:

- The same source is chosen for the ADC and the step input module to offset the inconsistence between the ADC reference and step input sources. The sample value of the ADC reflects the proportion of the response waveform in the high level of step input directly. Thus, the demands for power design decrease, and the calibration procedure is simplified. However, the proportion of variations in the single-sensing-coil structured response with distance in a high level of step input is limited.
- The distance measurement range of 0–1.0 mm is generally undetected quantitatively because of the mechanical manufacturing deviation. The distance measurement range of 1.0–5.0 mm is the major detection range. However, a nonlinear relationship exists between the sensitivity of the sensing coil and distance (Figure 6). The detection sensitivity in the secondary range of small distance is relatively high, whereas the detection sensitivity in the primary detection range of large distance is relatively low.

As illustrated in Figure 7, the single-sensing-coil structured response changes greatly with the distance at approximately 18.24 μS. At this moment, the variation in response waveform in the range of 0–7 mm is 738.7 mV (proportion in the dynamic range of the ADC is 22%). The variation in response waveform in the range of 1.0–7.0 mm is 273.6 mV (8.3%). In actual applications, only less than 8.3% dynamic range of ADC can be fully used, which restricts the sensitivity of the IPS.

A distant sensing coil is added on the single-sensing-coil structured IPS, which is assembled between the nearby sensing coil and the processing circuit. The distant sensing coil is far from the target. Thus, the influences of the inductance component on distance changes are smaller than those of the nearby sensing coil. Two symmetric driving circuits are designed for the nearby and distant sensing coils. Two symmetric responses on the measurement nodes are differenced and amplified by an INA, and then samples are collected. Accordingly, the proportion of response variations in the dynamic range of ADC is improved.

### 4.1. Linear Interpolation and Reduction in Dimension

**Figure 8:**
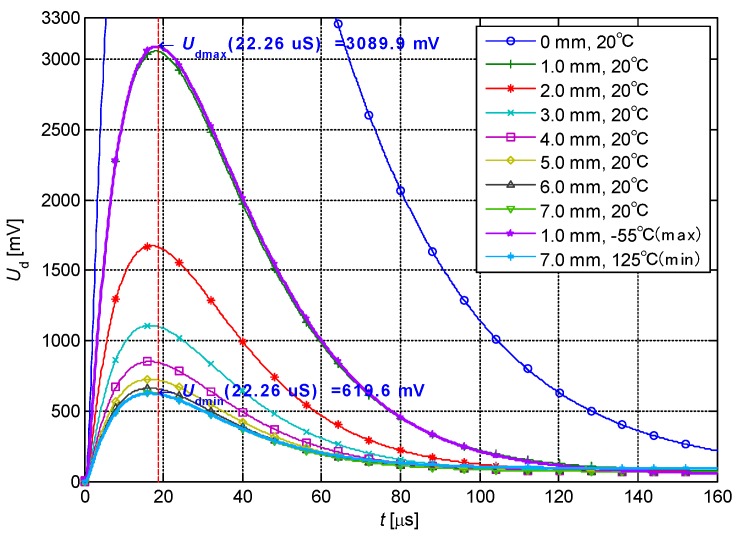
Response waveforms of the differential structured IPS.

The response of the differential structured IPS can be calculated from the response of the single-sensing-coil structured IPS. The proportion of differential signal after the amplification of the INA is significantly increased, where *U*_d_(*t*) = A(*U*_Cn_(*t*) − U_Cf_(*t*)), as shown in Figure 8. The sensitivity of the inductance component of the distant sensing coil decreases to 10% of that of the nearby sensing coil. In consideration of technological deviation, the deviations in resistance and inductance components between the nearby and distant sensing coils can be set to 5%. The length of cable is set as 20 m and gain A is 10. The response changes significantly with distance at approximately 22.26 μS. Under this circumstance, the variation in the sample is 2470.3 mV in the range of 1–7 mm (proportion in the dynamic range of the ADC is 75%). The response waveform has only one inflection point.

The length of cable is fixed, and only the resistance and inductance components in the single-sensing-coil structured IPS are variables. As confirmed in Reference [29], the sampling vector at two fixed moments and the vector impedance of the sensing coil form a functional relationship (Equation (15)). A two-dimensional look-up table method is constructed for computation.

The cable length is fixed and the resistance and inductance components are variables for the nearby and distant sensing coils in the differential structured IPS (Figure 3 and Equation (15)). The following conditions are also applied:

- The inductance components of two sensing coils are changed consistently with the distance.
- The resistance components of two sensing coils present similar variation with the environmental temperature.
- The differential structured response has one and only one inflection point, which is a monotone function after the inflection point. This characteristic is similar to that of the single-sensing-coil structured response.
- The differential structured IPS is composed of two single-sensing-coil structured IPSs, which are linear time-invariant (LTI) systems. The differential structured IPS composed by the two single-sensing-coil structured IPSs is also an LTI system.

A sample vector is obtained at two fixed moments after the inflection point of the differential structured response, which is inadequate for the characterization of four impedance parameters of two sensing coils. On the basis of constraints, a functional relationship between the environment variable (with two degrees of freedom for temperature and distance) and the two-dimensional sampling vector can be formed as follows:

(17) $$\left\{ \begin{array}{l} U_{d1}(D,T)=A(U_{\mathrm{Cn}}(t_{1},D,T)-U_{\mathrm{Cf}}(t_{1},D,T))\\ U_{d2}(D,T)=A(U_{\mathrm{Cn}}(t_{2},D,T)-U_{\mathrm{Cf}}(t_{2},D,T))\\ t_{2}>t_{1}>0 \end{array}\right.,$$

where *U*_d1_ and *U*_d2_ are the response values (sample values) at the two fixed moments, *D* is the distance between the IPS and the target, *T* is the environmental temperature, and A is the fixed gain. The relationship between the inductance component and *D* is constructed through calibration (Figure 6). The relationship between the resistance component and *T* can be set up through the temperature coefficient of resistance (3950 ppm/°C). On the basis of the look-up table method, the distance can be searched by the real-time sample vector (*U*_d1_, *U*_d2_).

The scale of the two-dimensional look-up table corresponding to the 14-bit (or higher accuracy) ADC is large. The proportion of sampling vector variation in the dynamic range of the ADC increases in the differential model. Thus, the effectiveness of the look-up table compression method, which depends on the defined range of sensing coil parameters, is limited. The dimension-reduced look-up table method realizes a dimensionality reduction in the two-dimensional look-up table through a linear approximation based on the approximate linear distribution between the sampling vector and temperature. This method can reduce storage and searching consumptions effectively.

After a sufficient delay, the differential structured response is close to the final response value, which can be considered as the complete discharging of equivalent inductance and distributed capacitance. The final response value is controlled by the resistive subdivision in the circuit, which does not correlate with the distance (inductance component). The second sample moment is set as “infinite” (>250 μS in this case). The constraint relationships of the sample vector (*U*_d1_, *U*_d2_) with *D* and *T* are separated. Equation (17) is simplified as

(18) $$\left\{ \begin{array}{l} U_{d1}(D,T)=A(U_{\mathrm{Cn}}(t_{1},D,T)-U_{\mathrm{Cf}}(t_{1},D,T))\\ U_{d2}(T)=A(U_{\mathrm{Cn}}(t_{2},T)-U_{\mathrm{Cf}}(t_{2},T))\\ t_{1}>0,t_{2}\to +\infty \end{array}\right..$$

In this case, the two-dimensional constraint (Equation (17)) between the sampling vector (*U*_d1_, *U*_d2_) and the environmental vector (*D*, *T*) is simplified into several constraints corresponding to *U*_d1_ and *D* at different values of *T*. The two-dimensional look-up table is divided into several lists of the table (indexes), which are only related to *T*. The contents of the table include one-dimensional look-up tables that record the relationship between *D* and *T*.

Here, *t*_1_ has an optimal value. If *t*_2_ corresponds to the final response value, then *t*_1_ is the only one effective sample time in the change process of the response waveform. Specifically, *t*_1_ is the moment of maximum change (with *D*) of the response waveform, and the highest detection resolution is achieved. The moment of maximum change of response waveform in Figure 8 is 22.26 μS. This moment is close to the inflection point of the response (Figure 8). Thus, *t*_1_ can be prolonged appropriately.

**Figure 9:**
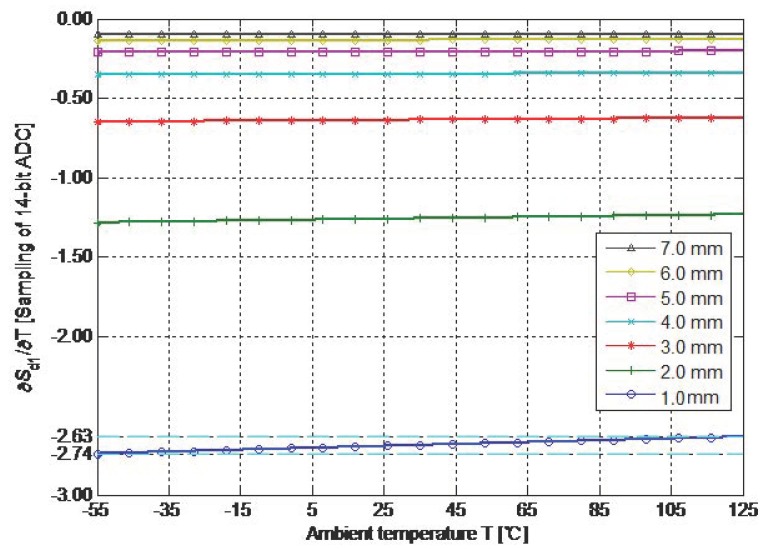
Nonlinear relationship between differential response and ambient temperature.

The relationship between *U*_d1_ and *D* is calibrated on every constant-temperature point, which cannot be realized in practice. Interpolation is performed by the approximate linear relationships of these relationships for reducing the times of constant-temperature calibration. In accordance with typical parameters, *t*_1_ is set as 25.00 μS and the length of cable is set as 20 m. *U*_d1_ is considered the function related to the independent variables *D* and *T*. The partial derivative ∂*U*_d1_(*D*, *T*)/∂*T* of the function *U*_d1_(*D*, *T*) about *T* is calculated. The partial derivatives that correspond to multi-group *D* are calculated and converted into the numerical values, which use the least significant bit (LSB) of the 14-bit ADC as a unit, as shown in Figure 9. The curves reflect the nonlinear relationships between the sample value (*S*_d1_) corresponding to different values of *D* and *T*. The curves in Figure 9 are approximate to the horizontal line (constant). Thus, *S*_d1_(*T*) is close to an oblique line, the nonlinear relationship is the most serious at the minimum *D*, and the accumulative (integral) deviation in the variation range of *T* is 0.11 LSB (of the 14-bit ADC). Therefore, an approximately linear relationship exists between *S*_d1_ and *T*, and the total deviation in the range of typical parameters can be neglected for the sample resolution of the 14-bit ADC.

The significance of the approximate linear relationship between the sample value *S*_d1_ and *T* is that the relationship between *S*_d1_ and *D* calibrated at two calibration temperatures can characterize the relationship in any ambient temperature. Specifically, the look-up tables corresponding to *S*_d1_ and *T* under any ambient temperature in the engineering range can be deduced from two sets of calibrations. In addition, the distance can be searched through the calibration table on the basis of the interpolation algorithm. Thus, the two-dimensional look-up table is simplified into two one-dimensional look-up tables.

### 4.2. Online Computation Flow

Different cable lengths and manufacturing deviations determine that the relationship between *S*_d1_ and *D* can be calibrated simultaneously. The calibration distance interval of 0.1 mm is taken as an example. In the range of 1–6 mm, each group of the one-dimensional look-up table contains the 61 sample values *S*_d1_ on the calibration distance points at the calibration temperatures.

**Figure 10:**
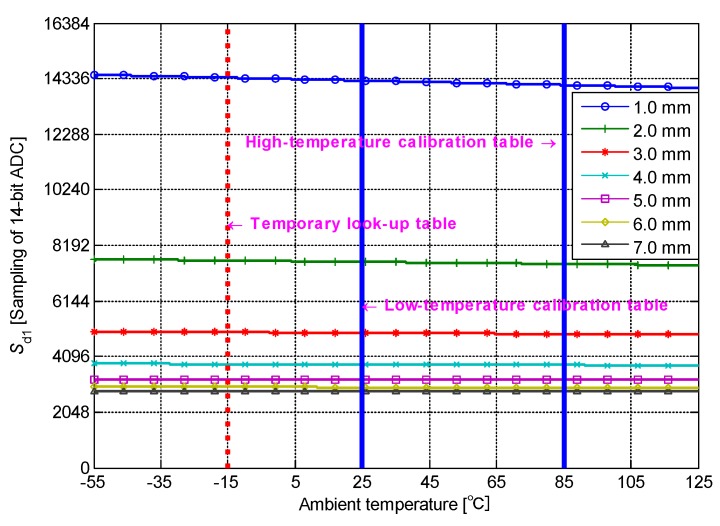
Process of calibration and calculation.

The relationship between the sample values *S*_d1_ and *D* is calibrated at two different fixed calibration temperatures. The high-temperature (85 °C) and low-temperature (25 °C) calibration tables correspond to the vertical coordinates of the focuses of two fixed calibration temperatures (solid vertical line in Figure 10) and 61 oblique lines of *S*_d1_(*T*). The two groups of 61 sample values (*S*_d1L_10_–*S*_d1L_70_ and *S*_d1H_10_–*S*_d1H_70_) and the corresponding (*S*_d2L_, *S*_d2H_) are recorded by a precise electronically controlled displacement platform at two calibration temperatures. This information is the complete calibration data. The sensor detection circuit acquires the real-time sample vector (*S*_d1_, *S*_d2_) by the ADC in the measurement process. At the interpolation temperature corresponding to *S*_d2_, the values of *S*_d1_ on the calibration distance points can be calculated from the high- and low-temperature calibration tables, that is, the temporary look-up table in Figure 10. The real-time relationship between *S*_d1_ and *D* at the measuring temperature is different from the relationship recorded in the high- and low-temperature calibration tables. An approximately linear relationship exists between *S*_d1_ and *T* (with only one corresponding relation with *S*_d2_). The proportional relationship between the sample value *S*_d2_ at the measuring temperature and the sample values (*S*_d2L_, *S*_d2H_) at the two calibration temperature determines the horizontal position of the temporary look-up table in Figure 10. On the basis of this proportional relationship and the two calibration tables, linear approximation is performed to obtain the temporary look-up table. The location of the real-time sample value *S*_d1_ in the temporary look-up table indicates the measured distance.

The temporary look-up table (*S*_d1T_i_) can be calculated by

(19) $$\left\{ \begin{array}{l} S_{d1T\_i}=(S_{d1H\_i}-S_{d1L\_i})\frac{S_{d2}-S_{d2L}}{S_{d2H}-S_{d2L}}+S_{d1L\_i}\\ i\;=\;10,11,12,\ldots ,70 \end{array}\right.,$$

where *S*_d2_ is the real-time sample value obtained in the measurement process, and *S*_d1L_i_, *S*_d1H_i_, *S*_d2L_, and *S*_d2H_ are the data of the high- and low-temperature calibration tables. The calculation to produce the temporary look-up table is an online calculation. The operator is introduced to simplify the online calculation as follows:

(20) $$\left\{ \begin{array}{l} S_{d1T\_i}=S_{d2}M_{di}+N_{di}\\ M_{di}=\frac{S_{d1H\_i}-S_{d1L\_i}}{S_{d2H}-S_{d2L}}\\ N_{di}=\frac{S_{d2H}S_{d1L\_i}-S_{d2L}S_{d1H\_i}}{S_{d2H}-S_{d2L}}\\ \;i\;=\;10,11,12,\ldots ,70 \end{array}\right.,$$

where M_i_ and N_i_ are calibration operators calculated from the high- and low-temperature look-up tables. In the calibration process, M_i_ and N_i_ are calculated offline and saved as the calibration operators. In the measurement process, the calculation that produces the temporary look-up table is simplified into one multiply–add operation between the real-time sample value *S*_d2_ and the calibration operators. This procedure simplifies the online calculation.

As shown in Table 1, the calibration data are composed of 61 groups of calibration operators M_i_ and N_i_. Furthermore, (*S*_2L_, *S*_2H_) in the original calibration data are mixed in the calibration operators M_i_ and N_i_ through calculation, which does not need to be recorded independently. The online detection and calculation procedures of IPS are listed as follows:

- Apply step input on the nearby and distant sensing coils.
- Acquire real-time sample vector (*S*_d1_, *S*_d2_) at two fixed delay moments.
- Substitute *S*_d2_ into Equation (17) for 61 iterations of a multiply–add operation to obtain the temporary look-up table (*S*_d1T_i_).
- Compare *S*_d1_ in the temporary look-up table bottom-up, and stop the search when *S*_d1_ is higher than data (*S*_d1T_i_) in the temporary look-up table.
- The integral part of the measured distance is the sum of calibrated step lengths for searching (and adding the starting point of calibration), which corresponds to *x* × 0.1 mm in the table.

Therefore, one differential structured IPS method based on a look-up table is proposed. The method has small storage and search consumption, simple online calculation, and saves the use of process control units, such as MCUs or DSPs.

## 5. Results and Discussion

**Figure 11:**
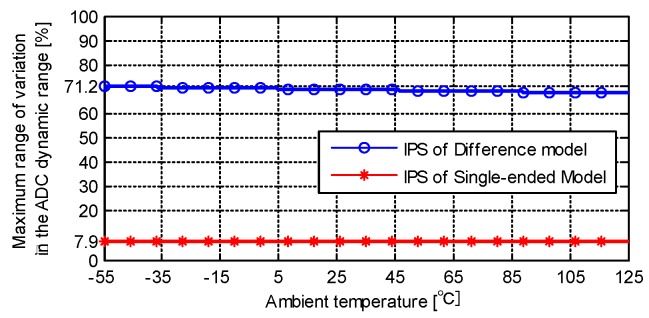
Maximum variation range in the analog−digital converter (ADC) dynamic range.

The direct advantage of the differential structured IPS is that the proportion of sample variation ranges with distance in the dynamic range of the ADC is high. The sample accuracy of the ADC is fully used. The maximum sample variation ranges of the single-sensing-coil structured IPS and the differential structured IPS are calculated using the typical parameters, as shown in Figure 11 (*t*_1_ = 25.00 μS, cable length = 20 m). The maximum variation range of sample changes slightly with the variation in environmental temperature. Given the same condition (1–7 mm), the maximum variation ranges of the single-sensing-coil structured IPS and differential structured IPS are 7.9% and 71.2% in the dynamic range of the ADC, respectively.

The full use of dynamic range of the ADC increases the detection sensitivity of the IPS. At different distances and temperatures, the detection sensitivities of the differential structured IPS and the single-sensing-coil structured IPS are shown in Table 2, where the unit is LSB/0.10 mm (*t*_1_ = 25.00 μS, cable length = 20 m). It represents the variable quantity (in LSB of the 14-bit ADC) of response corresponding to every 0.1-mm calibration distance interval at the corresponding conditions. The detection sensitivities of the two kinds of IPSs decrease slowly with the decrease in temperature, but increase significantly with the increase in distance. The maximum detection sensitivities are achieved at minimum temperature and distance. The value for the differential structured IPS is 1201.4 LSB/0.10 mm, and the value for the single-sensing-coil structured IPS is 135.5 LSB/0.10 mm. Under this condition, the detection sensitivity of the differential structured IPS is 8.8 times that of the single-sensing-coil structured IPS.

The application of linear approximation needs a small interpolation error to match with the increased detection sensitivity of the IPS. This condition is the key point of linear approximation. The high and low calibration temperatures are set as 100 °C and 20 °C to calculate the interpolation error between the differential structured IPS and the single-sensing-coil structured IPS at different distances and temperatures. The results are shown in Table 3. The interpolation errors (absolute value) of the two kinds of IPSs increase as the temperature gets far from the high and low calibration temperatures. The interpolation errors of the differential structured IPS are positive at the temperature between the high and low calibration temperature range, and are negative in other ranges. The sign distribution of interpolation errors of the differential structured IPS is opposite to that of the single-sensing-coil structured IPS. The interpolation errors (absolute value) of the differential structured IPS are negatively related to distance, which is contrary to that of the single-sensing-coil structured IPS. This feature is the key advantage of the differential structured IPS. Although some of the interpolation errors of the differential structured IPS are higher than those of the single-sensing-coil structured IPS (in the upper left and upper right of Table 2), the detection sensitivity of the differential structured IPS increases significantly with the decrease in distance (Table 2). This condition can offset the distance error caused by the increased interpolation error.

The interpolation error on distance is mapped in Table 4. The data distribution indicates that the detection sensitivity of the differential structured IPS increases significantly with the decrease in distance. This condition offsets the increase in interpolation error in LSB (absolute value) and decreases the distance error (absolute value) with the reduction in distance. The distribution of interpolation error in LSB (absolute value) of the single-sensing-coil structured IPS is contrary to that of the differential structured IPS. The distance error (absolute value) increases rapidly with the increase in distance because the proportion of the increased interpolation error (in LSB) in the reduced detection sensitivity is amplified, and the increase in distance error (absolute value) is accelerated. The maximum distance error of the single-sensing-coil structured IPS is 99.376 μm (−55 °C, 7.00 mm), and that of the differential structured IPS is −3.240 μm, which is only 3.3% of that of the single-sensing-coil structured IPS. The practical value increases significantly.

The differential structured measurement method requires two symmetric analog measurement modules, which increases the volume and power consumption of the sensor. It is actually the cost of the performance improvement of the differential structured IPS. The following key contributions are made:

1. The responses of two symmetric circuits are differenced and amplified by the INA, and the proportion of the variation in the dynamic range of ADC of the differential response increases significantly. The proportion of the variation increases from 8.3% to 75% compared with that of single-sensing-coil structured method, thereby realizing full use of the conversion ability of the high-accuracy ADC and increasing the sensitivity and resolution of the distance measurement.
2. The dimension of the two-dimensional look-up table decreases through the linear approximation based on the approximate linear distribution of the sampling vector and environmental temperature. This method solves the large-scaled look-up table problems brought by the high-accuracy ADC and decreases the consumption of storage and searching resources as follows:
  - The second sampling moment is set to “infinite”, and the constraint relationships of sampling vectors over distance and temperature are separated. The two-dimensional constraints are simplified as constraints over the sample value (*S*_d1_) and distance under different temperatures. The two-dimensional look-up table can be recorded as a group of one-dimensional look-up tables.
  - The relationship between the sample value (*S*_d1_) and temperature is approximately linear. The number of one-dimensional look-up tables can be decreased to two. In addition, the relationships between the sample value (*S*_d1_) and the distance under two calibration temperatures are set up by calibration. Specifically, the corresponding one-dimensional look-up tables under any temperature can be obtained from the two calibration tables through the interpolation algorithm while meeting the error conditions.
3. The increase in interpolation error is offset by the distribution of the measurement sensitivity, and the distance error decreases. At −55 °C and 7.0 mm, the maximum distance error of the differential structured IPS is decreased to 3.3% of that of the single-sensing-coil structured IPS. The practical value improves significantly.
4. The differential amplifier structure inhibits the common-mode signal, offsets influences of external electromagnetic interference on the symmetric response circuits effectively, and improves the electromagnetic compatibility of the IPS. The temperature effects on the symmetric response circuits of nearby and distant sensing coils are also weakened in the same environment.
5. The differential structured and dimension-reduced IPS method reduces storage, searching consumption and online computation complexity, and saves the use of process control components. The numerical decoupling calculation separates the resistance and inductance components of the IPS sensing coils and improves the temperature adaptation of IPS.

The proposed differential structured IPS is compared with those in publicly reported references, which are engaged in the same application background in the field of aviation and have similar parameters, as shown in Table 5. The differential structured IPS method calculates, separates, and extracts the mixed responses of the sensing coil to distance and environmental temperature. The look-up table method is simplified by linear approximation. These new ideas reflect the comparative advantages of the differential structured IPS in inhibiting the temperature drift of the sensing coil, thereby enhancing the detection sensitivity and decreasing the interpolation error.

## 6. Conclusions

A differential structured and dimension-reduced IPS method was proposed in this study on the basis of previous studies on the dimension-reduced look-up table method [56]. The proposed method assembled the distant and nearby sensing coils relative to the target, and these sensing coils formed two symmetric response circuits. The two responses were produced through synchronous excitation. After these signals were differenced and amplified by the INA, differential response was sampled by the ADC at two fixed moments. The sample values were processed by the new structural algorithm, and the distance between the IPS and the target was searched by the dimension-reduced look-up table method.

The measurement sensitivity could be further improved by increasing the INA gain at the cost of giving up the quantitative measurement of the small distance measurement range with low practical value in consideration of specific demands. This method provided flexibility of the design.

The differential structured and dimension-reduced IPS method designed in this work realized the numerical decoupling calculation of the vector impedance of sensing coils by using 61 look-up table units. The results of performance verification indicated that, compared with the single-sensing-coil structured IPS, the measuring resolution of the designed IPS method increased from 135.5 LSB/0.10 mm to 1201.4 LSB/0.10 mm, and the interpolation error decreased from 99.376 μm to −3.240 μm. This differential structured and dimension-reduced IPS method presented evident advantages compared with similar IPS methods in terms of measurement accuracy.

## 7. Patents

1. Shao Zhibiao, Guo Yixin. Aviation-specific displacement sensor measuring method: CN, ZL201210078266.9[E]. 02 Apr 2014. (authorized)
2. Shao Zhibiao, Guo Yixin. Measurement method for aviation-specific displacement sensor: US(PCT), US2015051857-A1[E]. 22 Sep 2014.
3. Shao Zhibiao, Guo Yixin. Data processing method of inductive type displacement sensor: CN, ZL201610278608.X[E]. 17 Jul 2018. (authorized)
4. Shao Zhibiao, Guo Yixin. Interference elimination method for inductance type displacement sensor: CN, ZL201710115059.9[E]. 31 May 2017. (authorized)

## Figures and Tables

**Table 1:** Storage, calculation, and search process of the look-up table.

Distance	Calibration Operator M	Calibration Operator N	Temporary Look-up Table *S*_d1T_i_	Sample Value *S*_d1_
1.0 mm	M_0_	N_0_	*S*_d__1T___10_	Less than *S*_d__1T___1__0_
1.1 mm	M_1_	N_1_	*S*_d__1T___11_	Less than *S*_d__1T___11_
1.2 mm	M_2_	N_2_	*S*_d__1T___12_	Less than *S*_d__1T___12_
…	…	…	…	Less than…
*x* × 0.1 mm	M_x_	N_x_	*S*_d__1T___x_	Greater than *S*_d__1T___x_
…	…	…	…	Greater than…
7.0 mm	M_70_	N_70_	*S*_d__1T___70_	Greater than *S*_d__1T___70_

**Table 2:** Comparison of measurement sensitivities. IPS—inductive proximity sensor; LSB—least significant bit.

Distance (mm)	Sensitivity of Differential Structured IPS/Single-Coil Structured IPS (LSB/0.10 mm)						
	−55 °C 9.29 Ω	−25 °C 10.85 Ω	5 °C 12.42 Ω	35 °C 13.98 Ω	65 °C 15.55 Ω	95 °C 17.11 Ω	125 °C 18.67 Ω
1.00–1.10	1201.4/135.5	1194.5/134.7	1187.5/133.8	1180.7/133.0	1173.8/132.2	1167.0/131.5	1160.3/130.7
2.00–2.10	443.2/49.4	440.2/49.1	437.2/48.7	434.2/48.4	431.2/48.0	428.3/47.7	425.4/47.4
3.00–3.10	184.5/20.5	183.2/20.3	181.9/20.2	180.5/20.0	179.2/19.9	177.9/19.8	176.6/19.6
4.00–4.10	86.4/9.6	85.8/9.5	85.2/9.4	84.5/9.4	83.9/9.3	83.3/9.2	82.6/9.2
5.00–5.10	41.6/4.6	41.3/4.6	41.0/4.5	40.6/4.5	40.3/4.5	40.0/4.4	39.7/4.4
6.00–6.10	23.3/2.6	23.1/2.6	22.9/2.5	22.8/2.5	22.6/2.5	22.4/2.5	22.2/2.5
7.00–7.10	11.7/1.3	11.6/1.3	11.5/1.3	11.4/1.3	11.3/1.3	11.2/1.2	11.1/1.2

**Table 3:** Distribution and comparison of interpolation errors.

Distance (mm)	Interpolation Errors of Differential Structured IPS/Single-Coil Structured IPS (LSB)						
	−55 °C 9.29 Ω	−25 °C 10.85 Ω	5 °C 12.42 Ω	35 °C 13.98 Ω	65 °C 15.55 Ω	95 °C 17.11 Ω	125 °C 18.67 Ω
1.00	−7.082/1.109	−3.401/0.534	−0.849/0.134	0.575/−0.091	0.922/−0.146	0.217/−0.034	−1.503/0.239
2.00	−3.275/1.218	−1.573/0.586	−0.392/0.147	0.266/−0.100	0.426/−0.160	0.100/−0.038	−0.695/0.262
3.00	−1.705/1.256	−0.819/0.604	−0.204/0.151	0.139/−0.103	0.222/−0.165	0.052/−0.039	−0.362/0.270
4.00	−0.992/1.271	−0.477/0.612	−0.119/0.153	0.081/−0.104	0.129/−0.167	0.030/−0.039	−0.211/0.274
5.00	−0.648/1.279	−0.312/0.615	−0.078/0.154	0.053/−0.105	0.085/−0.168	0.020/−0.040	−0.139/0.275
6.00	−0.472/1.282	−0.227/0.617	−0.057/0.154	0.039/−0.105	0.062/−0.168	0.015/−0.040	−0.101/0.276
7.00	−0.378/1.284	−0.182/0.618	−0.045/0.155	0.031/−0.105	0.050/−0.169	0.012/−0.040	−0.081/0.276

**Table 4:** Mapping of interpolation errors on distances.

Distance (mm)	Interpolation Errors of Differential Structured IPS/Single-Coil Structured IPS (μm)						
	−55 °C 9.29 Ω	−25 °C 10.85 Ω	5 °C 12.42 Ω	35 °C 13.98 Ω	65 °C 15.55 Ω	95 °C 17.11 Ω	125 °C 18.67 Ω
1.00	−0.589/0.819	−0.285/0.397	−0.071/0.100	0.049/−0.068	0.079/−0.110	0.019/−0.026	−0.130/0.183
2.00	−0.739/2.465	−0.357/1.195	−0.090/0.301	0.061/−0.206	0.099/−0.333	0.023/−0.079	−0.163/0.554
3.00	−0.924/6.127	−0.447/2.971	−0.112/0.749	0.077/−0.513	0.124/−0.829	0.029/−0.197	−0.205/1.379
4.00	−1.148/13.261	−0.556/6.431	−0.140/1.621	0.096/−1.110	0.154/−1.796	0.037/−0.427	−0.256/2.986
5.00	−1.559/27.742	−0.755/13.455	−0.190/3.391	0.130/−2.323	0.210/−3.757	0.050/−0.893	−0.349/6.249
6.00	−2.027/49.694	−0.983/24.103	−0.247/6.074	0.169/−4.161	0.274/−6.731	0.065/−1.600	−0.455/11.196
7.00	−3.240/99.376	−1.571/48.201	−0.396/12.148	0.271/−8.321	0.439/−13.461	0.104/−3.200	−0.730/22.391

**Table 5:** Comparison of the present study with related works. IC—integrated circuit; MCU—micro-controller unit; DSP—digital signal processor.

Performance	Present study	Single-coil structured IPS in previous study [56]	Application-specific IC [41]	Honeywell ZS-00305 [39]
Temperature Drift Reduction	Self-adaptive, compensation is unnecessary	Self-adaptive, compensation is unnecessary	Customized thermal resistance compensation is required	Unknown
Measurement Method	Analog–digital mixed	Analog–digital mixed	Analog	Analog
Quantitative Output	Yes	Yes	No	No
Cable Adaptability	Recalibration is unnecessary	Recalibration is needed	Analog parameters need to be adjusted	Unknown
Response Variation in Dynamic Range	71.2%	7.9%	None	None
Measurement Sensitivity	1201.4 LSB/0.10 mm	135.5 LSB/0.10 mm	None	None
Interpolation Distance Error	−3.240 μm	99.376 μm	None	None
Size of Look-up Table (units)	122	142	None	None
MCU or DSP	Non-adoptive	Non-adoptive	Non-adoptive	Non-adoptive
